# Adjuvant treatment with dexamethasone plus anti-C5 antibodies improves outcome of experimental pneumococcal meningitis: a randomized controlled trial

**DOI:** 10.1186/s12974-015-0372-y

**Published:** 2015-08-15

**Authors:** E. Soemirien Kasanmoentalib, Mercedes Valls Seron, B. Paul Morgan, Matthijs C. Brouwer, Diederik van de Beek

**Affiliations:** Center for Immunity and Infection (CINIMA): Department of Neurology, Center for Immunity and Infection (CINIMA), Academic Medical Center, University of Amsterdam, PO Box 22660, 1100DD Amsterdam, The Netherlands; Institute of Infection and Immunity, Cardiff University School of Medicine, Cardiff, Wales UK

**Keywords:** Pneumococcal meningitis, Randomized controlled trial, Dexamethasone, Complement component C5, Monoclonal antibody, Animal models

## Abstract

**Background:**

We compared adjunctive treatment with placebo, dexamethasone, anti-C5 antibodies, and the combination of dexamethasone plus anti-C5 antibodies in experimental pneumococcal meningitis.

**Methods:**

In this prospective, investigator-blinded, randomized trial, 96 mice were infected intracisternally with 10^7^ CFU/ml *Streptococcus pneumoniae* serotype 3, treated with intraperitoneal ceftriaxone at 20 h, and randomly assigned to intraperitoneal adjunctive treatment with placebo (saline), dexamethasone, anti-C5 antibodies, or dexamethasone plus anti-C5 antibodies. The primary outcome was survival during a 72-h observational period that was analyzed with the log-rank test. Secondary outcome was clinical severity, scored on a validated scale using a linear mixed model.

**Results:**

Mortality rates were 16 of 16 mice (100 %) in the placebo group, 12 of 15 mice (80 %) in the dexamethasone group, 25 of 31 mice (80 %) in the anti-C5 antibody group, and 18 of 30 mice (60 %) in the dexamethasone plus anti-C5 antibody group (Fisher’s exact test for overall difference, *P* = .012). Mortality of mice treated with dexamethasone plus anti-C5 antibodies was lower compared to the anti-C5 antibody-treated mice (log-rank *P* = .039) and dexamethasone-treated mice (log-rank *P* = .040). Clinical severity scores for the dexamethasone plus anti-C5 antibody-treated mice increased more slowly (0.199 points/h) as compared to the anti-C5 antibody-treated mice (0.243 points/h, *P* = .009) and dexamethasone-treated mice (0.249 points/h, *P* = .012). Modeling of severity data suggested an additive effect of dexamethasone and anti-C5 antibodies.

**Conclusion:**

Adjunctive treatment with dexamethasone plus anti-C5 antibodies improves survival in severe experimental meningitis caused by *S. pneumoniae* serotype 3, posing an important new treatment strategy for patients with pneumococcal meningitis.

## Background

Mortality and morbidity rates are high among adults with acute bacterial meningitis, especially those with pneumococcal meningitis [1]. Unfavorable neurologic outcomes are not the result of treatment with inappropriate antimicrobial agents, since cerebrospinal fluid cultures are sterile 24 to 48 h after the start of antibiotic therapy [2]. Studies in animals have shown that bacterial lysis, induced by treatment with antibiotics, leads to inflammation in the subarachnoid space, which may contribute to an unfavorable outcome [2]. These studies also show that adjuvant treatment with anti-inflammatory agents, such as dexamethasone, reduces both cerebrospinal fluid inflammation and neurologic sequelae [2].

This knowledge has prompted many clinical trials on the effect of dexamethasone in acute bacterial meningitis. A Cochrane meta-analysis showed that adjunctive dexamethasone treatment did not reduce mortality in children with bacterial meningitis but did decrease hearing loss from 20 % in the control group to 15 % in corticosteroid-treated children (risk ratio (RR) 0.74; 95 % CI 0.62–0.89) [3]. For adults with community-acquired bacterial meningitis, the results of a European controlled trial showed that adjunctive dexamethasone, given before or with the first dose of antibiotic therapy, was associated with a reduction in mortality (7 vs. 15 %; RR 0.48; 0.24–0.96) [4]. This beneficial effect was most obvious in adults with pneumococcal meningitis, in whom the mortality rate decreased from 34 to 14 %.

Novel treatment strategies are needed to further improve prognosis of bacterial meningitis. In a nationwide genetic association study, we found that complement activation plays a crucial role in the outcome of patients with pneumococcal meningitis [5]. We showed that inhibition of complement component C5 with monoclonal antibodies decreased mortality in a pneumococcal meningitis mouse model [5]. In this study, mice were inoculated with serotype 2 pneumococci, an uncommon serotype in patients [1]. Differences in virulence between pneumococcal serotypes may affect the efficacy of adjunctive treatments [6]. Furthermore, anti-C5 antibody treatment was not administered together with dexamethasone, which is currently the standard therapy in pneumococcal meningitis [7]. Finally, in recent years, new standards for animal experiments have been proposed to improve the predictive value of preclinical (animal) research [8]. These included randomization, blinding, sample size calculations, and standards for data handling in animal experiments in a similar fashion to protocols routine in clinical trials.

The aims of the current study were (1) to validate the beneficial effect of anti-C5 antibodies in a pneumococcal meningitis mouse model using a common pneumococcal serotype [1, 9] and (2) to assess the potential added benefit of combining anti-C5 antibody treatment with adjunctive dexamethasone in a randomized investigator-blinded trial.

## Methods

A well-characterized mouse model of pneumococcal meningitis was used in this study [10]. Nine- to 11-week-old male C57BL/6NCrl mice (Charles River Laboratories, Germany) were injected with 1 μl of 10^7^ CFU/ml *Streptococcus pneumoniae* serotype 3 (ATCC 6303; American Type Culture Collection, Rockville, MD, USA) into the cisterna magna under short-term isoflurane anesthesia.

We first evaluated whether the expression profile of the terminal complement complex (C5b-9) was similar in mice infected with serotype 3 compared to previous observations in a serotype 2 model. Mice were inoculated and sacrificed at 6 h (*n* = 12) and 24 h (*n* = 12) after infection. Mice inoculated with sterile saline and sacrificed at 24 h (*n* = 6) served as control. After euthanasia, mice were perfused with sterile phosphate-buffered saline, the brain was removed and the left hemisphere was taken up in 20 % weight per volume sterile saline and homogenized with a tissue homogenizer. Bacterial titers were determined by plating serial 10-fold dilutions on sheep-blood agar plates and incubating for 16 h at 37 °C. Subsequently, brain homogenates were lysed in lysis buffer (150 mM NaCl, 15 mM Tris, 1 mM MgCl_2,_ 1 mM CaCl_2_, 1 % Triton, 4 μl/ml AEBSF, 50 μg/ml EDTA-Na_2_, 10 ng/ml pepstatin, 10 ng/ml leupeptin, pH 7.4) on ice for 20 min and centrifuged at 3600 rpm at 4 °C for 15 min. The supernatant was stored at −20 °C until assayed. C5b-9 levels were determined in brain homogenates by ELISA (USCN Life Science).

Subsequently, we performed a randomized investigator-blinded trial. All mice were treated intraperitoneally 20 h after infection with a single dose of ceftriaxone (100 mg/kg) in combination with adjuvant treatment. Adjuvant treatment was administered once together with the antibiotics and consisted of sterile saline (*n* = 16; “placebo group”), dexamethasone (0.5 mg/kg, *n* = 16), a neutralizing antibody against C5 (clone BB5.1; 1 mg/mouse, *n* = 32), or dexamethasone plus anti-C5 antibodies in previously mentioned doses (*n* = 32); groups were randomized in a ratio 1:1:2:2. To compare anti-C5 antibody-treated mice with non-anti-C5 antibody-treated mice and dexamethasone-treated mice with non-dexamethasone-treated mice, we aimed to detect a decrease in mortality from 80 to 50 %. Using an 80 % power, one-sided testing, and significance level of *P* < .05, we needed 32 mice per combined group. Since we were most interested in the comparison between the anti-C5 antibody-treated group and the dexamethasone plus anti-C5 antibody-treated group, we randomized in a 1:1:2:2 ratio, in which twice as many mice are randomized for the anti-C5 antibody group and dexamethasone plus anti-C5 antibody group compared to the placebo- and dexamethasone-treated groups.

All mice received identical total amount of fluids. Mice were randomly assigned to treatment groups using a computer-generated random number list (Microsoft Excel 2010), and all researchers were blinded for treatment allocation. The randomization code was broken after the last experiment was finished. All animals were clinically examined and scored prior to inoculation, directly following inoculation and at regular intervals during the 72-h observation period. The following clinical features were scored: weight loss, activity, time to return to upright position, state of fur, posture, eye discharge or protrusion, respiration rate, irregular/labored breathing, epilepsy, limb paresis, and coordination [9]. A score of 0 indicates healthy mice, and mice with a score of 15 or more were terminally ill and therefore euthanized. Humane endpoints other than the score of 15 or more at which the mice were euthanized were >25 % weight loss, ≥2 seizures per 15 min, status epilepticus, and hemi-paralysis. Mice were euthanized by intraperitoneal injection of ketamine (190 mg/kg, Eurovet Animal Health, Bladel, the Netherlands) in combination with dexmedetomidine (0.3 mg/kg, Pfizer Animal Health, Capelle aan den Ijssel, the Netherlands). The experiments were performed in three tranches; all treatment groups were represented in these tranches. The number of colony-forming units (CFUs) in inoculates was determined for each experiment by serial dilutions on sheep-blood agar plates. Animal experiments were approved by the Institutional Animal Care and Use Committee of the Academic Medical Center Amsterdam.

The Mann-Whitney *U* test was used to compare C5b-9 levels between groups. In the randomized investigator-blinded trial, survival was analyzed using a log-rank test. To model the clinical scores of these mice, several (non)linear mixed models were compared using the likelihood ratio test statistic (LRTS) or Akaike’s A information criterion (AIC). A linear mixed model with a random slope and fixed effects for dexamethasone (D) and/or anti-C5 antibody treatment, time, and the interactions between dexamethasone and time and anti-C5 antibody treatment and time modeled the data the best (fixed effects: D + anti-C5 antibodies + Time + D*Time + anti-C5 antibodies*Time). Differences were considered significant at a *P* value of <.05.

## Results

**Fig. 1:**
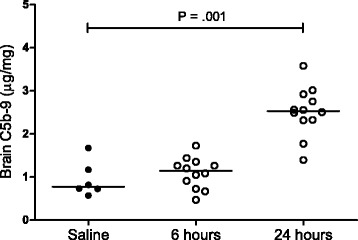
C5b-9 is expressed in brain during experimental pneumococcal meningitis. C5b-9 (μg/mg) protein levels in brain of WT mice inoculated with sterile saline (*n* = 6) or 10^7^ CFU/ml of *S. pneumoniae* serotype 3 (*n* = 24) and sacrificed at 6 or 24 h post inoculation. Mice with pneumococcal meningitis showed increased brain C5b-9 levels compared to saline-inoculated mice at 24 h after inoculation (median 0.78 vs. 2.53 μg/mg tissue, Mann-Whitney *U* test *P* = .001). *Lines* represent median values

Bacterial meningitis was confirmed in all mice infected with *S. pneumoniae* serotype 3 by determination of CFU in brain homogenates, showing similar bacterial titers per time-point (data not shown). C5b-9 levels were determined in brain homogenates of these 30 mice. Mice with pneumococcal meningitis showed increased brain C5b-9 levels compared to saline-inoculated mice at 24 h after inoculation (median 0.78 vs. 2.53 μg/mg tissue, *P* = .001; Fig. 1).

In the randomized investigator-blinded trial, 96 C57BL/6NCrl male mice were injected into the cisterna magna with *S. pneumoniae* serotype 3. The number of CFUs in inoculates was similar between tranches (1.21 × 10^7^, 0.94 × 10^7^, 1.22 × 10^7^ CFU/ml). Four mice showed a limb paresis directly following inoculation and were subsequently taken out of the experiment and euthanized. All remaining mice showed signs of illness 20 h after infection and were randomly assigned to the four treatment groups: 16 mice to the placebo group, 15 mice to the dexamethasone group, 31 mice to the anti-C5 antibody group, and 30 mice to the dexamethasone plus anti-C5 antibody group. All mice were treated intraperitoneally with ceftriaxone.

**Fig. 2:**
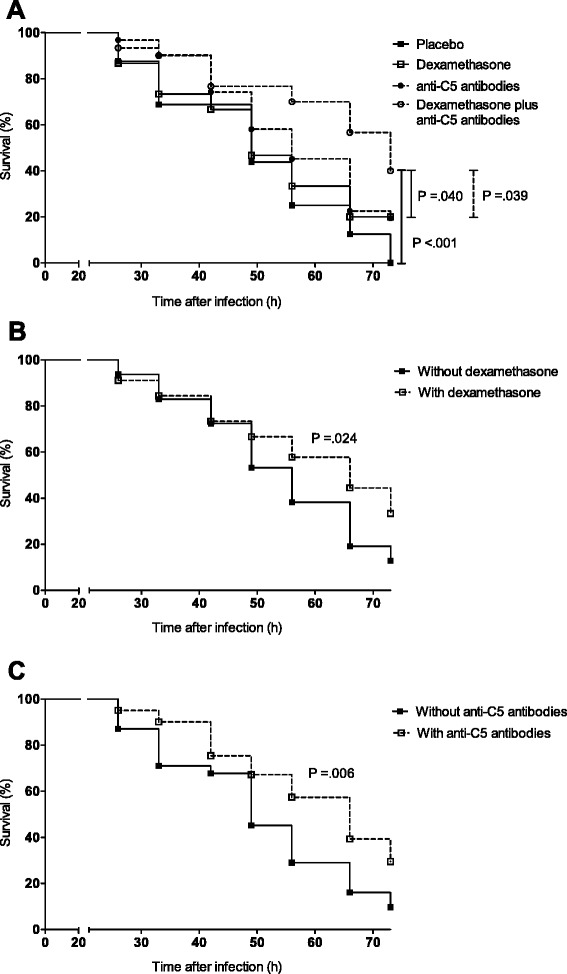
Adjuvant treatment with dexamethasone plus anti-C5 antibodies reduces mortality in experimental pneumococcal meningitis. Kaplan-Meier survival curves of WT mice inoculated with 10^7^ CFU/ml of *S. pneumoniae* serotype 3 and treated at 20 h after infection with ceftriaxone (100 mg/kg) in combination with adjuvant treatment. Adjuvant treatment consisted of sterile saline (*n* = 16; “placebo group”), dexamethasone (0.5 mg/kg, *n* = 15), a neutralizing antibody against C5 (C5-Ab; 1 mg/mouse; clone BB5.1, *n* = 31), or dexamethasone plus anti-C5 antibodies in previously mentioned doses (*n* = 30). **a** Adjuvant treatment with dexamethasone plus anti-C5 antibodies resulted in a significant reduction in mortality compared to placebo (log-rank *P* < .001), anti-C5 antibodies, (log-rank *P* = .039), and dexamethasone (log-rank *P* = .040). **b** Adjuvant dexamethasone significantly reduced mortality when all treatments with dexamethasone were compared to all treatments without dexamethasone (log-rank *P* = .024). **c** Treatments with anti-C5 antibodies significantly reduced mortality when compared to all treatments without anti-C5 antibodies (log-rank *P* = .006)

The first animals reached an endpoint at 26 h after infection, and the overall mortality rate during the 72-h observation period was 71 of 92 (77 %). The mortality rates were 16 of 16 mice (100 %) in the placebo group, 12 of 15 mice (80 %) in the dexamethasone group, 25 of 31 mice (80 %) in the anti-C5 antibody group, and 18 of 30 mice (60 %) in the dexamethasone plus anti-C5 antibody group (Fig. 2a; Fisher’s exact test for overall difference, *P* = .012). The combination of dexamethasone and anti-C5 antibodies resulted in a significant reduction in mortality compared to placebo (log-rank *P* < .001), anti-C5 antibodies (log-rank *P* = .039), and dexamethasone (log-rank *P* = .040). Adjuvant dexamethasone significantly reduced mortality when all treatments with dexamethasone were compared to all treatments without dexamethasone (mortality rate 67 vs. 87 %, log-rank *P* = .024; Fig. 2b). Treatments with anti-C5 antibodies significantly reduced mortality when compared to all treatments without anti-C5 antibodies (mortality rate 71 vs. 90 %, log-rank *P* = .006; Fig. 2c). Clinical severity scores for the dexamethasone plus anti-C5 antibody-treated mice increased slowly as compared to dexamethasone-treated mice (0.199 vs. 0.249 points/h, *P* = .012), anti-C5 antibody-treated mice (0.199 vs. 0.243 points/h, *P* = .009), and placebo-treated mice (0.199 vs. 0.293 points/h, *P* = .001). When analyzing dexamethasone vs. no dexamethasone treatment, a slower increase of the clinical score with time was observed (reduction 0.044 ± 0.018 points/h, *P* = .013). This effect was independent of anti-C5 antibody treatment that also caused a slower increase of the clinical score with time (reduction 0.050 ± 0.019 points/h, *P* = .009) compared to no anti-C5 antibody treatment. A model with inclusion of a D*C5*Time interaction did not significantly improve fitting of the data (*P* = .317). This indicates that the effects of dexamethasone and anti-C5 antibody treatments on clinical score are additive with no synergistic enhancement.

## Discussion

The results of our randomized study show that adjunctive treatment with dexamethasone plus anti-C5 antibodies is beneficial in experimental pneumococcal meningitis. Adjunctive treatment with dexamethasone plus anti-C5 antibodies led to lower clinical severity scores and improved survival. The observed effects of this combined adjunctive therapy were superior to that observed in animals adjunctively treated with dexamethasone or anti-C5 antibodies alone. Since anti-C5 antibodies are currently licensed for clinical use (Eculizumab™) and other anti-C5 agents are in clinical trials [11–14], our results present a promising treatment option for future patients with community-acquired pneumococcal meningitis, although the current treatment costs are an important limiting factor for future studies and implementation.

We previously demonstrated that a common variant in complement component 5 was associated with unfavorable outcome in adults with community-acquired pneumococcal meningitis [5]. The anaphylatoxin C5a was identified as the crucial complement product in pneumococcal meningitis. Deficiency of the receptor for C5a led to an improved clinical status and clinical course in mice. C5a receptor deficiency and C5 neutralization resulted in a marked reduction of CSF WBC counts in the pneumococcal mouse model, with lower concentrations of IL-6, CXCL1, and CXCL2. Pretreatment with CXCL1 and CXCL2 antibodies caused a reduction of CSF WBC count, but to a lesser extent than that found in *C5ar1*^−/−^ mice, indicating that C5a regulates chemokine expression but also has a direct chemotactic effect. Neutralization experiments showed that adjunctive treatment with anti-C5 antibodies improved outcome in mice with pneumococcal meningitis. However, these experiments were performed as a proof-of-concept, not according to the new standards for animal experiment [8]. In these experiments, infection was induced using *S. pneumoniae* serotype 2, a very uncommon cause of bacterial meningitis [1]. We have now showed the therapeutic effect of neutralizing antibodies against C5 in a randomized controlled manner, using *S. pneumoniae* serotype 3, a common pneumococcal serotype [1, 9].

Our results show a beneficial effect of adjunctive dexamethasone on clinical severity and survival. Dexamethasone has become routine in patients with pneumococcal meningitis as result of clinical trials and meta-analyses [3, 4, 15–18]. The mechanisms by which dexamethasone inhibits inflammation are not clear, but it decreases pro-inflammatory cytokine production in monocytes, dendritic cells, astroglial cells, and neutrophils, increases the production of anti-inflammatory cytokines such as IL-10, inhibits ROS production by leukocytes, and decreases leukocyte adherence [2]. Dexamethasone also acts on multiple molecules of the TLR downstream signaling cascade [2]. A nationwide implementation of dexamethasone therapy reduced the nationwide mortality rate of adult pneumococcal meningitis from 30 to 20 % in the Netherlands [9].

In the current study, we investigated the additive effect of these two adjunctive anti-inflammatory therapies, dexamethasone and anti-C5 antibodies. We used a randomized investigator-blinded trial with large group sizes to investigate this new therapeutic strategy in experimental pneumococcal meningitis. To our knowledge, for the first time, we show an effect of adjunctive therapy on clinical course and survival in experimental pneumococcal meningitis. Nevertheless, our study has several limitations, mainly limiting our external validity towards the human situation. First, antibiotic treatment was only given once at 20 h, during an observation period of 72 h, while in clinical practice, patients are treated twice a day with ceftriaxone; further, mice were treated with a single dose of dexamethasone, while dexamethasone is given four times daily for 4 days in patients [3, 7, 18, 19]. These dosing regimens, chosen to keep the treatment regimen comparable to the previous published experiment [5], can be regarded as common practice in animal studies but may have led to a underestimation of the effect of dexamethasone. We observed a higher mortality than expected in the experiments (50 % expected vs. 60 % observed in the treatment group, 80 % expected vs. 100 % observed in the placebo group) which may have contributed to the significance of the results. The severity of pneumococcal meningitis varies between experiments, which can be caused by variation in bacterial virulence.

Differences between pneumococcal serotypes may affect the efficacy of anti-C5 antibody treatment. We showed that inhibition of complement component C5 improves outcome in serotypes 2 and 3 pneumococcal meningitis [5], suggesting the effect of anti-C5 antibodies is serotype independent. However, for serotypes other than 2 and 3, data are currently lacking on anti-C5 antibody treatment. We chose serotype 3 for our mouse model as it was the most common serotype in a clinical cohort performed in the Netherlands [1, 10]. Although following introduction of conjugate vaccines, the proportion of cases due to serotype 3 has decreased, it is still among the most common serotypes in adult pneumococcal meningitis [9, 20].

The effect of anti-C5 antibody treatment in bacterial meningitis caused by pathogens other than *S. pneumoniae* is unclear and should be studied separately. Whereas dexamethasone was shown to be of no harm in meningococcal meningitis, complement inhibition may be detrimental, as the role of complement was found to be substantially different between pneumococcal and meningococcal meningitis [21, 22].

In conclusion, in a randomized investigator-blinded study, we observed a strong protective effect of adjunctive treatment with dexamethasone plus anti-C5 antibodies. Our data provide further evidence for the efficacy of complement inhibition in pneumococcal meningitis, supporting the need for a phase II clinical trial to evaluate the effect of combined adjunctive dexamethasone plus C5-antibody treatment in patients with pneumococcal meningitis.
